# The status of insecticide resistance of *Anopheles coluzzii* on the islands of São Tomé and Príncipe, after 20 years of malaria vector control

**DOI:** 10.1186/s12936-024-05212-6

**Published:** 2024-12-18

**Authors:** Maria Correa, Janete Lopes, Carla A. Sousa, Gustavo Rocha, Robin Oriango, Andreia Cardetas, Joao Viegas, Anthony J. Cornel, Gregory C. Lanzaro, João Pinto

**Affiliations:** 1 Vector Genetics Laboratory, Department of Pathology, Microbiology, and Immunology, University of California, Davis, CA USA; 2 Global Health and Tropical Medicine, LA-REAL, Instituto de Higiene e Medicina Tropical, Universidade Nova de Lisboa, Lisbon, Portugal; 3 Programa Nacional de Eliminação do Paludismo, Centro Nacional de Endemias, São Tomé, São Tomé and Príncipe; 4 Mosquito Control Research Laboratory, Department of Entomology and Nematology, University of California, Parlier, CA USA

**Keywords:** Malaria, *Anopheles coluzzii*, Insecticide resistance, *kdr* mutations, São Tomé and Príncipe

## Abstract

**Background:**

Insecticide-based malaria vector control has been implemented on the islands of São Tomé and Príncipe (STP) for more than 20 years. During this period malaria incidence was significantly reduced to pre-elimination levels. While cases remained low since 2015, these have significantly increased in the last year, challenging the commitment of the country to achieve malaria elimination by 2025. To better understand the reasons for increasing malaria cases, levels and underlying mechanisms of insecticide resistance in the local *Anopheles coluzzii* populations were characterized.

**Methods:**

Mosquito larval collections were performed in the rainy and dry seasons, between 2022 and 2024, in two localities of São Tomé and one locality in Príncipe. Susceptibility to permethrin, α-cypermethrin, pirimiphos-methyl and DDT was assessed using WHO bioassays and protocols. Intensity of resistance and reversal by PBO pre-exposure were determined for pyrethroid insecticides. The *kdr* locus was genotyped by PCR assays in subsamples of the mosquitoes tested.

**Results:**

*Anopheles coluzzii* populations were fully susceptible to pirimiphos-methyl, but high levels of resistance to pyrethroids and DDT were detected, particularly in São Tomé rainy season collections. Increasing the pyrethroid and DDT dosages to 5 $\times$ and 10 $\times$ did not restore full susceptibility in all populations. Pre-exposure to PBO resulted into partial reversal of the resistance phenotype suggesting the presence of cytochrome P450 oxidases-mediated metabolic resistance. The L1014F knockdown resistance mutation was present in *An. coluzzii* on both islands but at much higher frequency in São Tomé where it was associated with the resistant phenotype.

**Conclusions:**

Future vector control interventions should consider the use of non-pyrethroid insecticides or combination with synergists to overcome the high levels of pyrethroid resistance. Alternative control methods not dependent on the use of insecticides should be additionally implemented to achieve malaria elimination in STP.

**Supplementary Information:**

The online version contains supplementary material available at 10.1186/s12936-024-05212-6.

## Background

Several attempts to reduce the malaria burden on the Islands of São Tomé and Príncipe (STP) have been made throughout the years. In the early 1980’s, a malaria eradication program using biannual indoor residual spraying (IRS) of dichloro-diphenyl-trichloroethane (DDT) and weekly prophylaxis with chloroquine reduced malaria prevalence from 19.2 to 0.6% by 1982 [1]. After abrupt cessation of malaria control, malaria incidence rose to epidemic proportions in 1985–1986 with high case-mortality rates that were attributed to combinations of financial constraints and vector resistance to DDT [1]. High caseloads continued as chloroquine was solely used for treatment resulting in rise of resistance to this antimalarial drug [2].

Starting in 2004, scale-up interventions designed to achieve millennium development goals for malaria were implemented in STP. These included IRS with α-cypermethrin, pyrethroid impregnated long lasting insecticidal nets (LLIN), artemisinin-amodiaquine and artemether-lumefantrine as first-line and second-line drugs for treatment of uncomplicated malaria, and intermittent preventive therapy with sulfadoxine-pyrimethamine [3]. An evaluation after three years of this scaled-up intervention demonstrated remarkable success by decreasing patient consultations, hospitalizations and deaths by more than 85%, 80%, and 95%, respectively, in all age groups, resulting in an overall parasite prevalence of 2.1% and leading Príncipe island to pre-elimination levels [3, 4].

In 2021, STP was selected by the World Health Organization (WHO) as a priority country for malaria elimination under the E-2025 initiative [5]. This meant reinforced financial investment and technical support commitments to improve vector control with measures such as (i) rotational use of active ingredients (α-cypermethrin, malathion, pirimiphos-methyl and clothianidin) for IRS, (ii) distribution of new generation LLIN impregnated with α-cypermethrin and, more recently a mosaic of α-cypermethrin and piperonyl butoxide (PBO), and (iii) widespread larval control with *Bacillus thuringiensis israelensis*. In a report from the Ministry of Health, malaria incidence and mortality decreased a further 47% from 2010 to 2015 (unpublished data), but since then overall malaria incidence has remained constant [6]. Recently though, in the first 12 weeks of 2024, official data from the National Malaria Elimination Programme show a doubling of cases compared to the same 12 weeks in 2022 and 2023. Because of this increase, the STP Ministry of Health has expressed challenges precluding the achievement of malaria elimination by 2025, including a lower compliance of residents with preventative measures such as allowing IRS access and LLIN use and possibly, insecticide resistance.

Presently, the vector of malaria in STP is the FOREST cytoform of *Anopheles coluzzii* [7–9]. In contrast to mainland populations, this mosquito displays marked exophagic, exophilic and zoophilic behaviors, possibly due to environmental reasons (*e.g.* house construction) and the effect of indoors-based vector control [10–12]. *Anopheles coustani*, the only other anopheline in the archipelago, is restricted to the Island of São Tomé, where it is much less common than *An. coluzzii* and its role as a vector of malaria is currently unknown [7, 8]*.*

An important consideration challenging malaria control is the issue of insecticide resistance. Several countries in Africa have reported resistance to the four classes of insecticides (pyrethroids, organophosphates, carbamates and organochlorines) mostly used in public health in at least one malaria vector species and high intensity resistance to pyrethroids has been detected more frequently in west Africa than in other subregions [13]. Two point mutations at position 1014 of the voltage-gated sodium channel gene, resulting in the substitution of a leucine residue by a phenylalanine (L1014F or *kdr-w*) or a serine (L1014S or *kdr-e*) are considered primary mutations conferring resistance to pyrethroids and DDT [14, 15]*.* Haplotype data suggests that the *kdr-w* allele arose multiple times in *Anopheles gambiae s.s.* and underwent a selective sweep across populations [16–18]. This mutation introgressed into *An. coluzzii* through hybridization with *An. gambiae s.s.* in specific locations and then selectively swept across West and Central African populations [19–21]. The *kdr-e* mutation has also selectively swept through populations of both species but also may have arisen independently [16, 17]. In addition to target-site mutations, insecticide resistance is also governed by metabolic mechanisms involving detoxifying enzyme superfamilies [22]. Of these, cytochrome P450 oxidases play a central role in resistance to pyrethroids [23], the major insecticide class used for bed net treatment.

In this study, we investigated current and past knockdown resistance genotypic frequencies and levels of insecticide resistance in *An. coluzzii* from STP in order to: (1) assess the status of resistance to pyrethroid and non-pyrethroid insecticides; (2) determine mechanisms underlying the resistance phenotypes, if present.

## Methods

### Mosquito collections

São Tomé and Príncipe islands have a long rainy season, from October to May, and a short dry season, from June to September. The long rainy season is interrupted by a second dry period, usually in January. To account for seasonal variations, larval collections were performed in both rainy and dry seasons, by standard sampling methods using dippers and pipettes in two localities of São Tomé island and one in Príncipe island, as illustrated in Fig. S1, Supplementary Materials.

In São Tomé, collections were carried out in Água Grande district and Ribeira Afonso in July–September 2022 (dry season) and again in May–June 2023 (rainy season). The district of Água Grande corresponds to the capital city of the country (São Tomé) and is characterized by an urban setting where over 60% of the *ca.* 220,000 inhabitants of São Tomé island live [24, 25]. The village of Ribeira Afonso harbors *ca.* 2,000 inhabitants living mainly from fishery and subsistence agriculture. In Príncipe, collections took place in St. António in July 2023 (dry season) and January 2024 (rainy season). This is the only urban centre of the island, where more than 80% of the *ca.* 9,000 people of this island live.

Collected larvae were reared to adults in trays filled with water from which they were collected and daily fed with *ca.* 10 mg of Koi fish food (Doctors Foster and Smith Koi Staple Diet, Rhinelander, WI USA). Water levels in the trays were kept constant by adding mineral water. Pupae were picked daily, and every 3 days’ worth of pupae were placed in a cubic cage (30 $\times$ 30 $\times$ 30 cm), in an insectary set at 27 ºC, 80% relative humidity and 12:12 h light: dark cycle. Emerged adults were provided constant access to 10% sucrose solution until 12 h before insecticide bioassay.

### Bioassays

Insecticide susceptibility to permethrin (0.75%), α-cypermethrin (0.05%), pirimiphos-methyl (0.25%) and dichloro-diphenyl-trichloroethane (DDT) (4%) was tested by WHO tube tests and protocols [26, 27]. A minimum of 100 three to five days-old non-blood fed females were exposed for one hour on WHO tubes with treated paper (diagnostic dose of each insecticide) along with 50 control specimens on WHO tubes with control papers (insecticide carrier). Mortality was recorded 24 h after exposure. When mortality in control tubes was between 5 and 20%, mortality in test tubes was corrected using Abbott’s formula. The population was deemed resistant if mortality was below 90%, suspected resistant if mortality was between 90 and 98%, and susceptible if mortality was above 98% [26].

Whenever resistance was detected for pyrethroids (*i.e.* permethrin and α-cypermethrin) intensity of resistance was tested by exposing mosquitoes to 5 $\times$ and 10 $\times$ the diagnostic dose [26]. In addition, presence of metabolic resistance mediated by monooxygenase enzymes was assessed by exposing mosquitoes to the synergist piperonyl butoxide (PBO) prior to insecticide exposure, following the WHO (2022) [28] protocol. PBO restores susceptibility by inhibiting the monooxygenases of the resistant mosquito.

In addition to the bioassays performed in 2022–2024, the results of two unpublished surveys carried out in Água Grande district, in April 2003 (rainy season) and September 2004 (dry season) are presented. WHO bioassays and procedures [29] were used to test permethrin (0.75%) and DDT (4%) at the diagnostic doses only. Non-blood fed females were used in the tests, but these mosquitoes were collected by landing catches performed with WHO aspirators between 20.00 and 00.00 h, so that mosquito age was not controlled in the assays.

### Genotyping of the knockdown resistance locus

Genomic DNA was extracted from pyrethroid susceptible, resistant and control individual mosquitoes using the peqGOLD Blood and Tissue DNA Mini Kit (VWR Avantor, Radnor PA, USA). Mosquitoes were identified to species of the *An. gambiae* complex by a PCR–RFLP assay targeting species-specific polymorphisms at the intergenic spacer of the ribosomal DNA [30]. Reactions were performed on 10 µL volume containing 1 µL (~ 1.4 ng) of DNA template.

For genotyping of the *kdr* locus, two allele-specific PCR assays were employed using the primers described by Martinez-Torres et al*.* [14] for the L1014F *kdr-w* mutation, and Ranson et al*.* [15] for the L1014S *kdr-e* mutation. Each PCR reaction was made in a volume of 15 µL containing 1 µL (~ 1.4 ng) of DNA template, 0.1 mM of primers Agd1 and Agd2, 0.4 mM of primers Agd3 (or Agd5) and Agd4, and 1 $\times$ Taq Plus master Mix (VWR Avantor, Radnor PA). The PCR cycling conditions consisted of an initial step of 5 min at 94 ºC followed by 35 cycles each with 30 s at 94 ºC, 40 s at 48ºC (Agd3) or 55 ºC (Agd5) and 40 s at 72 ºC, and a final 10 min extension at 72 ºC. Amplified products were visualized under UV light after separation by electrophoresis in 1.5% agarose gels stained with GreenSafe Premium (NZYTech, Lisbon, Portugal).

### Data analysis

Whenever mortality rates were between 90 and 98% a replicate of the diagnostic exposures was performed [26], and the value considered was the simple mean between replicates. Significant differences between groups were assessed by chi-squared tests on contingency tables or by the non-parametric equivalent Fisher’s exact test.

## Results

The results of susceptibility assays conducted at diagnostic doses for the four insecticides tested are shown in Table 1. In all tests, there was 100% mortality to pirimiphos-methyl indicating full susceptibility to this insecticide. In contrast, resistance to DDT and pyrethroids was found in most cases with mortality rates < 90%. In general, susceptibility was higher in Príncipe island (St. António) when compared with the two localities of São Tomé island (Água Grande and Ribeira Afonso). There was also a consistent trend for higher levels of susceptibility during the dry season when compared to the rainy season. These seasonal differences were significant in all cases where data was available for comparison (Table 1).

**Table 1:** *An. coluzzii* mortality rates at diagnostic doses in samples collected in São Tomé and Príncipe

		São Tomé Island						Príncipe Island		
		Água Grande			Ribeira Afonso			St. António		
		*N*	%	*p*	*N*	%	*p*	*N*	%	*p*
$\alpha$-cypermethrin	Dry	122	100.0	< 0.001	225	97.3*	< 0.001	225	92.3*	0.003
	Rainy	102	80.2		117	47.9		221	85.1*	
Permethrin	Dry	242	87.6*	< 0.001	102	98.1	< 0.001	–	–	N.A
	Rainy	109	60.6		108	73.2		104	85.6	
Pirimiphos-methyl	Dry	115	100.0	N.A	116	100.0	N.A	111	100.0	N.A
	Rainy	116	100.0		118	100.0		114	100.0	
DDT	Dry	218	90.5*	0.007	108	100.0	< 0.001	110	99.1	1.000**
	Rainy	112	79.9		116	79.3		108	100.0	

*N*: number of female mosquitoes exposed to the insecticide. %: percentage mortality 24 h after insecticide exposure; *average of two replicate experiments when the first exposure gave a mortality rate between 90 and 98% [34]. *p*: *p*-value of a chi-square test on contingency table with two degrees of freedom comparing dry and rainy season mortalities. ** Two-sided Fisher’s test

**Fig. 1:**
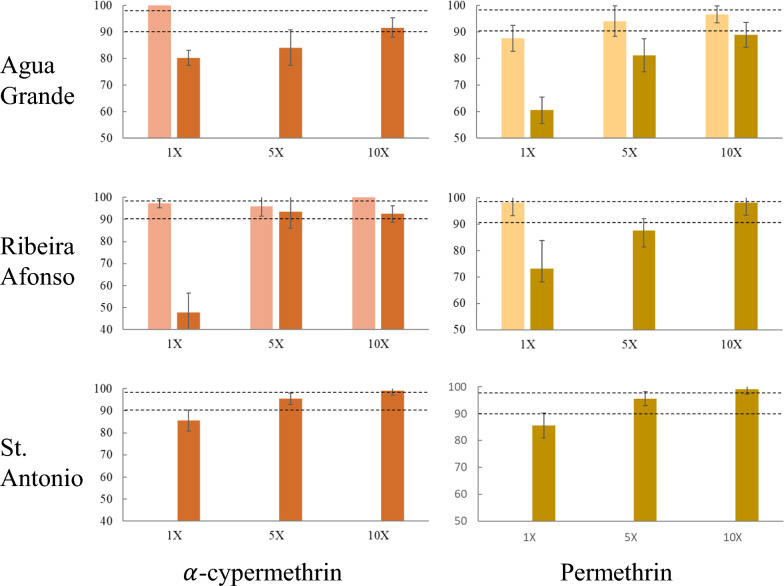
Intensity of resistance to pyrethroids in *Anopheles coluzzii* from São Tomé and Príncipe. Bar graphs display mortality rates at 1 $\times$, 5 $\times$ and 10 $\times$ the diagnostic dose for $\alpha$-cypermethrin (left) and permethrin (right) at the three localities surveyed. Y-axis: percent mortality, X-axis: dose exposure. In each graph the light-colored columns correspond to bioassay results conducted during the dry season and the dark columns refer to rainy season results. Intensity tests were not run in St. Antônio during the dry season. Error bars: 95% confidence intervals. Dashed lines indicate 98% and 90% thresholds for susceptibility and resistance, respectively

**Fig. 2:**
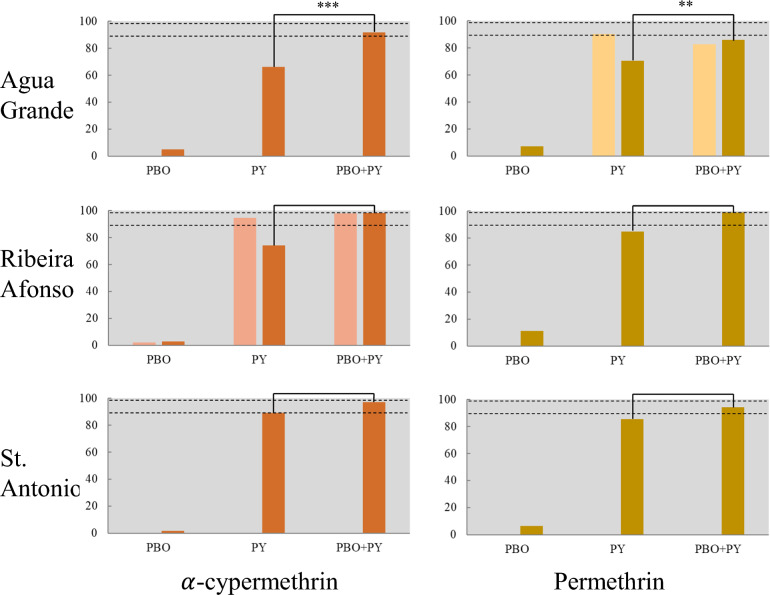
Synergistic effect of piperonyl butoxide on pyrethroid susceptibility in *Anopheles coluzzii* from São Tomé and Príncipe. Bar graphs display mortality rates 24 h after exposure to piperonyl butoxide 4% only (PBO), to the diagnostic dose of the pyrethroid insecticide only (PY), and to exposure to PBO 4% followed by exposure to the pyrethroid diagnostic dose (PBO + PY). Y-axis: percent mortality, X-axis: type of exposure. Light colored columns correspond to bioassays conducted in the dry season and dark columns are rainy season bioassays. Dashed lines indicate 98% and 90% thresholds for susceptibility and resistance, respectively. Square brackets show significant chi-square tests comparing mortality with and without PBO exposure; *p $<$ 0.05, **p $<$ 0.01, ***p $<$ 0.001

Increasing the dosage of pyrethroids to 5 $\times$ and 10 $\times$ the diagnostic dose resulted in increased mortality but not always sufficient to reach susceptibility levels (*i.e.*, $>$ 98%) (Fig. 1). This was the case for α-cypermethrin in Água Grande and Ribeira Afonso, and for permethrin in Água Grande, where mortality at 10 $\times$ the diagnostic exposure varied between 89 and 93%. Whenever comparisons were available (*i.e.*, Água Grande/permethrin; Ribeira Afonso/α-cypermethrin), mortality rates at 5 $\times$ and 10 $\times$ were consistently lower in the rainy season when compared to the dry season (Fig. 1). Pre-exposure to PBO significantly increased mortality to pyrethroids in samples collected in the rainy season, but complete reversal to susceptibility was only seen in Ribeira Afonso for both pyrethroids (Fig. 2). Differences between PBO + pyrethroid and pyrethroid only were non-significant for the two assays carried out in the dry season.

**Fig. 3:**
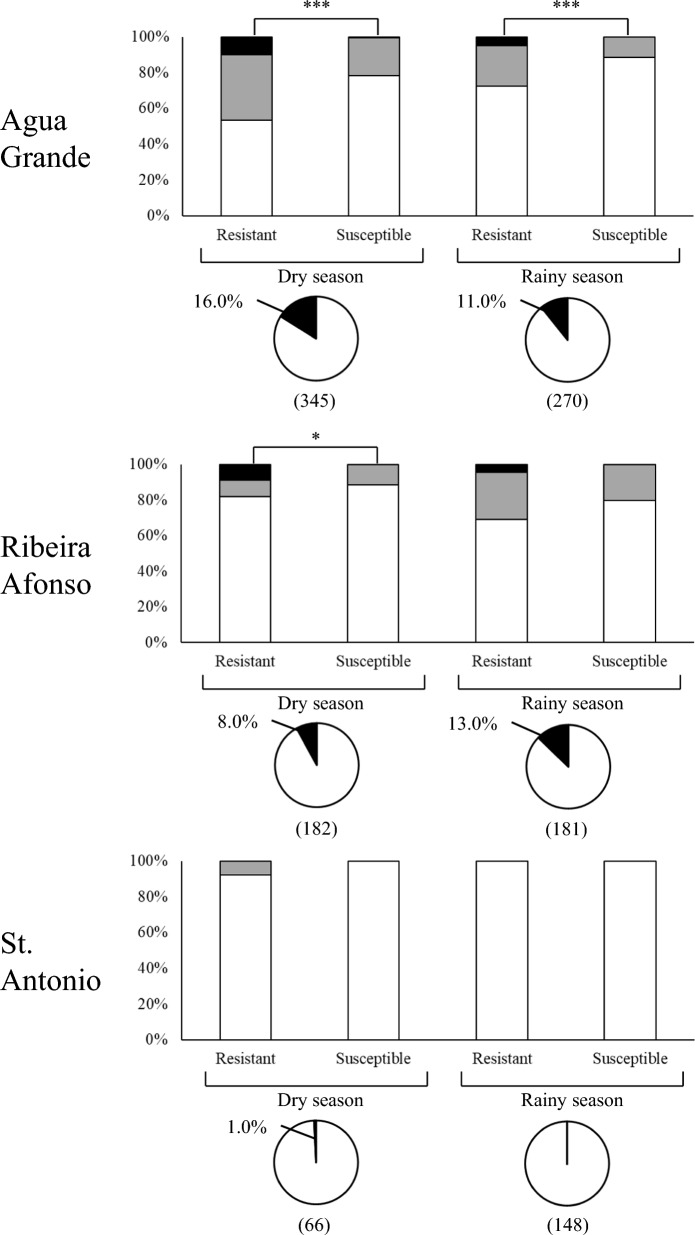
Knockdown resistance mutations in *Anopheles coluzzii* from São Tomé and Príncipe. Stacked bar graphs display the relative frequency (in percentage) of *kdr* genotypes in resistant and susceptible individuals exposed to pyrethroids or DDT in bioassays performed during the dry and rainy season in the three localities studied. White: *kds* (TTA/TTA) homozygotes; gray: *kds-kdr-w* (TTA/TTT) heterozygotes; black: *kdr-w* (TTT/TTT) homozygotes. Square brackets show significant chi-square tests comparing genotypes in susceptible and resistant mosquitoes *p $<$ 0.05, ***p $<$ 0.001. Pie charts refer to the relative frequency of each allele. White: *kds* (TTA); black: *kdr-w* (TTT). Sample sizes are in parenthesis

Figure 3 shows the results of the genotyping of the *kdr* locus. The L1014F (*kdr-w*) mutation was the only one found and it was detected in the three localities albeit at varying frequency, being highest in Água Grande, intermediate in Ribeira Afonso and lowest in St. António. In Água Grande, the frequency of *kdr-w* was higher in the dry season compared to the rainy season (chi-square test, p = 0.024). In contrast, Ribeira Afonso showed a higher, though not statistically significant, *kdr-w* frequency in the rainy season (chi-square test, p = 0.105). In Água Grande, in both seasons, there was a significant association between the resistance phenotype and the presence of the *kdr* allele (Fig. 3). This association was not so evident in Ribeira Afonso, where a marginally significant association was detected in the dry season only.

The comparison of current susceptibility levels to DDT and permethrin found in Água Grande with those recorded in 2003 and 2004, suggests a slight but steady decrease of mortality rates from 2004 onwards (Fig. S2A, Supplementary Materials). No *kdr* mutations were found in 2003 (*N* = 303) or 2004 (*N* = 100) compared to the 16% and 11% frequency of the *kdr-w* allele found in 2022 and 2023, respectively (Fig. S2B, Supplementary Materials).

## Discussion

High levels of resistance to pyrethroids were found in *An. coluzzii*, particularly in São Tomé island. Mortalities below 90% were obtained in exposures to 10 $\times$ the diagnostic dose. While this may be considered a predictor of operational failure, the real impact of the insecticide resistant phenotype in vector control remains uncertain [26, 31, 32]. The high levels of pyrethroid resistance found in STP may be a consequence of the intensive usage of this insecticide class for malaria control over the past 20 years in the country [3, 33, 34]. Permethrin has been used in STP since the 1990s in periodical campaigns of bed nets impregnation. In 2004, two additional pyrethroids, deltamethrin and α-cypermethrin, were introduced and widely used in the country with the establishment of LLIN [35]. In addition, α-cypermethrin was the insecticide of choice for IRS in the National Strategic Plan to Control Malaria implemented in 2004, being used for two consecutive years [33].

When comparing the two islands, pyrethroid resistance was consistently lower in Príncipe island. São Tomé island, and Água Grande district in particular, concentrates over 90% of the *ca.* 220.000 inhabitants of the country. Furthermore, malaria prevalence has been consistently lower in Príncipe and this region has implemented since 2023 a regime of reactive indoor spraying as opposed to the large-scale IRS employed in São Tomé [6, 7, 36]. These differences may explain a lower insecticide selective pressure in Príncipe when compared to São Tomé island.

The presence of the L1014F *kdr-w* mutation coupled with partial reversal of the resistant phenotype when mosquitoes were pre-exposed to PBO suggest that pyrethroid resistance in *An. coluzzii* from STP is mediated by both target-site and metabolic mechanisms. The inhibition of cytochrome P450 oxidase activity by PBO and the consequent increase in mortality after exposure to permethrin or α-cypermethrin suggests a role of this detoxifying enzyme superfamily in the resistance phenotype. This type of metabolic resistance has been reported in mainland populations of *An. coluzzii*, including in nearby West African countries, often with the concomitant presence of *kdr* mutations [37–39]. *CYP6M2*, *CYP6Z2* and *CYP9K1* are among the cytochrome P450 oxidase genes mostly associated with pyrethroid resistance in this vector species [23, 38]. Further studies involving gene expression analysis will be required to ascertain the specific genes involved in the resistance phenotype of *An. coluzzii* in STP.

The pyrethroid resistance-associated L1014F *kdr* mutation was found in both islands albeit at higher frequency in São Tomé island, where positive associations between *kdr-w* and the resistant phenotype were found. This result agrees with other studies that show increasing frequencies of resistance-associated alleles and the development of resistance after deployment of insecticide-based vector control [40, 41]. In STP, the *kdr-w* allele was not detected in collections performed in 2003 and 2004 suggesting a recent emergence of this resistance-associated allele. The first record of the *kdr-w* allele was in 2010 in São Tomé island, six years after the implementation of IRS with α-cypermethrin. In Príncipe this mutation was reported for the first time in 2014 [33].

However, there is no information about genotyping of *kdr* mutations between 2004 and 2010 and the first report showed that the *kdr-w* mutation was already present in 4 districts of São Tomé island in 2010 [33]. The emergence of the *kdr-w* allele in STP may be a result of mutational events or introgression with *kdr-*carrying mosquitoes that may have accidently reached the islands. Both origins, including interspecific introgression, have been previously described throughout the African continent [16–20]. Further studies involving haplotype analysis of genomic regions surrounding the *kdr* locus would help clarify the origin of the *kdr-w* allele in STP.

Haplotype diversity analysis has also shown evidence for strong positive selection of the *kdr-w* allele in West Africa, which may explain the rise of this allele from 0.3% to 54% in *An. coluzzii* from Ghana [18]. However, such an increase was not so evident in *An. coluzzii* from STP. The initial report of Chen et al*.* [33] describes *kdr-w* frequencies of 13% in 2010, reaching a peak in 2013 with 78% and decreasing to 40% in 2016. A second report shows *kdr-w* frequencies between 8 and 44% in three districts of São Tomé island sampled in 2018 [42]. These values contrast with the *kdr-w* frequencies found in this study (2022–2024) that did not exceed 16%. A decrease in *kdr-w* frequency may be explained by a relaxation of the selective pressure due to the interruption of pyrethroid-based IRS campaign in 2021 when a rotation scheme with non-pyrethroid insecticides started to be employed for IRS. It may also reflect a possible increase of the outdoor biting and resting preferences of this mosquito vector on the islands leading to a lower selective pressure [10, 11, 33].

Another important aspect was the seasonal differences in susceptibility levels, with increased resistance in the rainy season. Sampling bias in the dry season associated with a lower number of breeding sites might contribute to these differences. This would be the case if higher resistance were observed in the dry season, suggesting that more siblings carrying *kdr* alleles (or other resistance mechanisms) were used in bioassays. However, the differences found between seasons in *kdr* genotype distribution were only marginally significant in Água Grande and do not seem to explain the observed phenotypic patterns. Another explanation may be attributed to seasonal differences in the physiological/nutritional status of *An. coluzzii*. During the rainy season, the greater availability of larval habitats suitable for mosquito breeding reduces inter- and intraspecific competition leading to a higher nutritional intake and resulting in adults with an average higher percentage of body fat tissue, known to be rich in detoxifying enzymes [43, 44]. This hypothesis needs confirmation as it contrasts with previous observations that dry season mosquitoes are more resistant than rainy season ones [45, 46].

The higher resistance levels found in the dry season are generally explained by the selective pressure of pollutants in larval habitats that lead to increased expression of detoxifying enzymes such as P450 oxidases [46]. This is more evident in rural areas with marked seasonality where agriculture insecticide usage occurs mainly during the dry season. This may not be the case for São Tomé, in which the short duration of the dry season may not be sufficient to promote a high concentration of xenobiotics in larval habitats.

Regarding non-pyrethroid insecticides, resistance to DDT was also observed in São Tomé (but not in Príncipe). This agrees with the historical use of DDT during the malaria eradication programme in the 1980s and the emergence of resistance to this insecticide has been considered a major reason for disruption of this programme [1, 24]. This may also reflect cross-resistance, as DDT shares with pyrethroids the same active site, the voltage-gated sodium channel, and the L1014F mutation confers resistance to both insecticide classes [47, 48].

*Anopheles coluzzii* populations were fully susceptible to pirimiphos-methyl. This result may be explained by the fact that pirimiphos-methyl was used only in one campaign in the country (2019), and subsequently implemented in the rotation scheme, which appears to work positively to prevent resistance from arising [34]. This result contrasts to that observed for pyrethroids when widely used for consecutive years. While mechanisms of organophosphate resistance are likely to be absent from these island populations, this may also reflect lower selective pressure associated with the outdoors behavior of this mosquito in STP.

## Conclusion

The high levels of insecticide resistance found, specifically in São Tomé island and during the rainy season, reflect the selective pressures exerted by the extensive usage of pyrethroid insecticides for malaria vector control and raises concern about the efficacy of insecticide-based measures that are being implemented for malaria control. Recently, malaria cases have been increasing at a concerning pace in STP which may reflect a reduced efficacy of pyrethroid-based vector control. When planning future vector control interventions, selection of non-pyrethroid insecticides employed in rotation, and application of vector control at specific times of the year should be taken into account. In addition, alternative non-insecticidal malaria control tools should be considered in the malaria control strategic plan of the country.

## Supplementary Information

Supplementary material 1: Fig. S1. Map of São Tomé and Príncipe showing collection sites in São Tomé island and Príncipe island. Fig. S2. Temporal variation of insecticide resistance and knockdown resistance mutations in São Tomé and Príncipe.

Supplementary material 2: Fig. S2.

## Data Availability

No datasets were generated or analysed during the current study.

## Abbreviations

WHO: World Health Organization
IRS: Indoor residual spraying
LLIN: Long lasting insecticidal nets
DDT: Dichloro-diphenyl-trichloroethane
PBO: Piperonyl butoxide
Kdr: Knockdown resistance
*Bti*: *Bacillus thuringiensis israelensis*
PCR–RFLP: PCR-Restriction Fragment Length Polymorphism
