# Supplementary figures and images for: The status of insecticide resistance of Anopheles coluzzii on the islands of São Tomé and Príncipe, after 20 years of malaria vector control
Source: Malar J. 2024 Dec 18;23:390. doi: 10.1186/s12936-024-05212-6 (PMC11657776; doi:10.1186/s12936-024-05212-6)

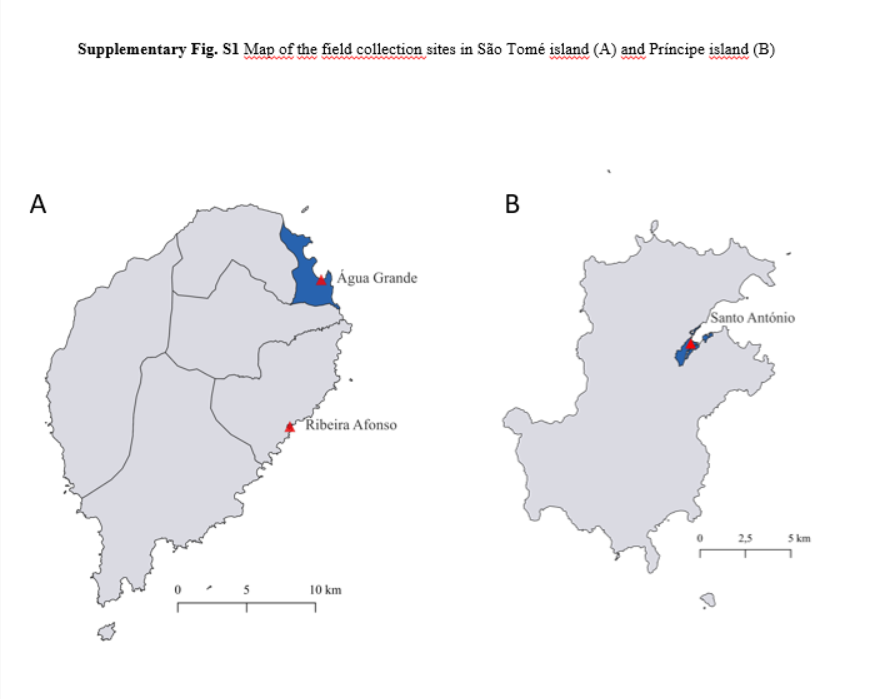


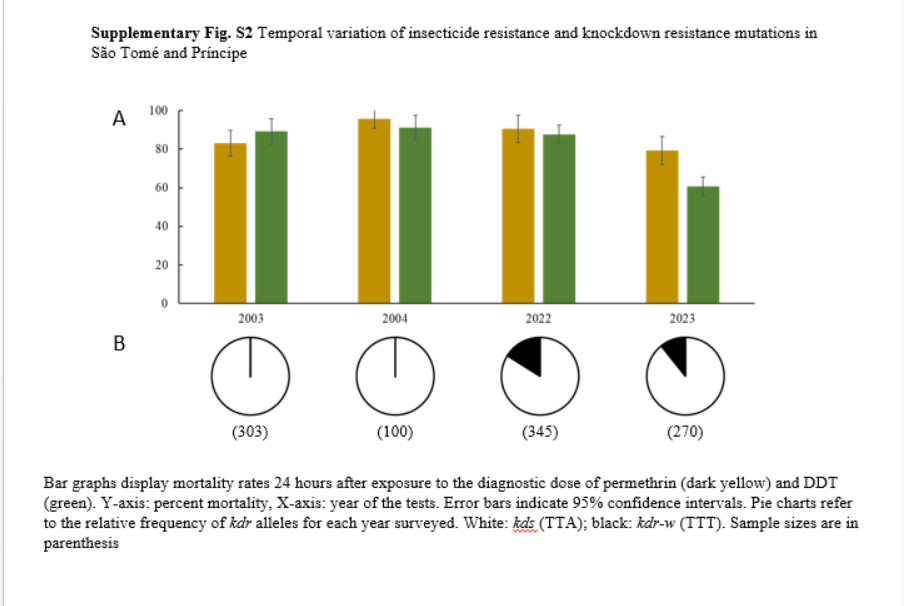

Supplement: Supplementary file 1 — Supplementary material 1: Fig. S1. Map of São Tomé and Príncipe showing collection sites in São Tomé island and Príncipe island. Fig. S2. Temporal variation of insecticide resistance and knockdown resistance mutations in São Tomé and Príncipe. [file 12936_2024_5212_MOESM1_ESM.docx]
