# Supplementary material for: The status of insecticide resistance of Anopheles coluzzii on the islands of São Tomé and Príncipe, after 20 years of malaria vector control
Source: Malar J. 2024 Dec 18;23:390. doi: 10.1186/s12936-024-05212-6 (PMC11657776; doi:10.1186/s12936-024-05212-6)
Supplement: Supplementary file 2 — Supplementary material 2: Fig. S2. [file 12936_2024_5212_MOESM2_ESM.pptx]

## Slide 1
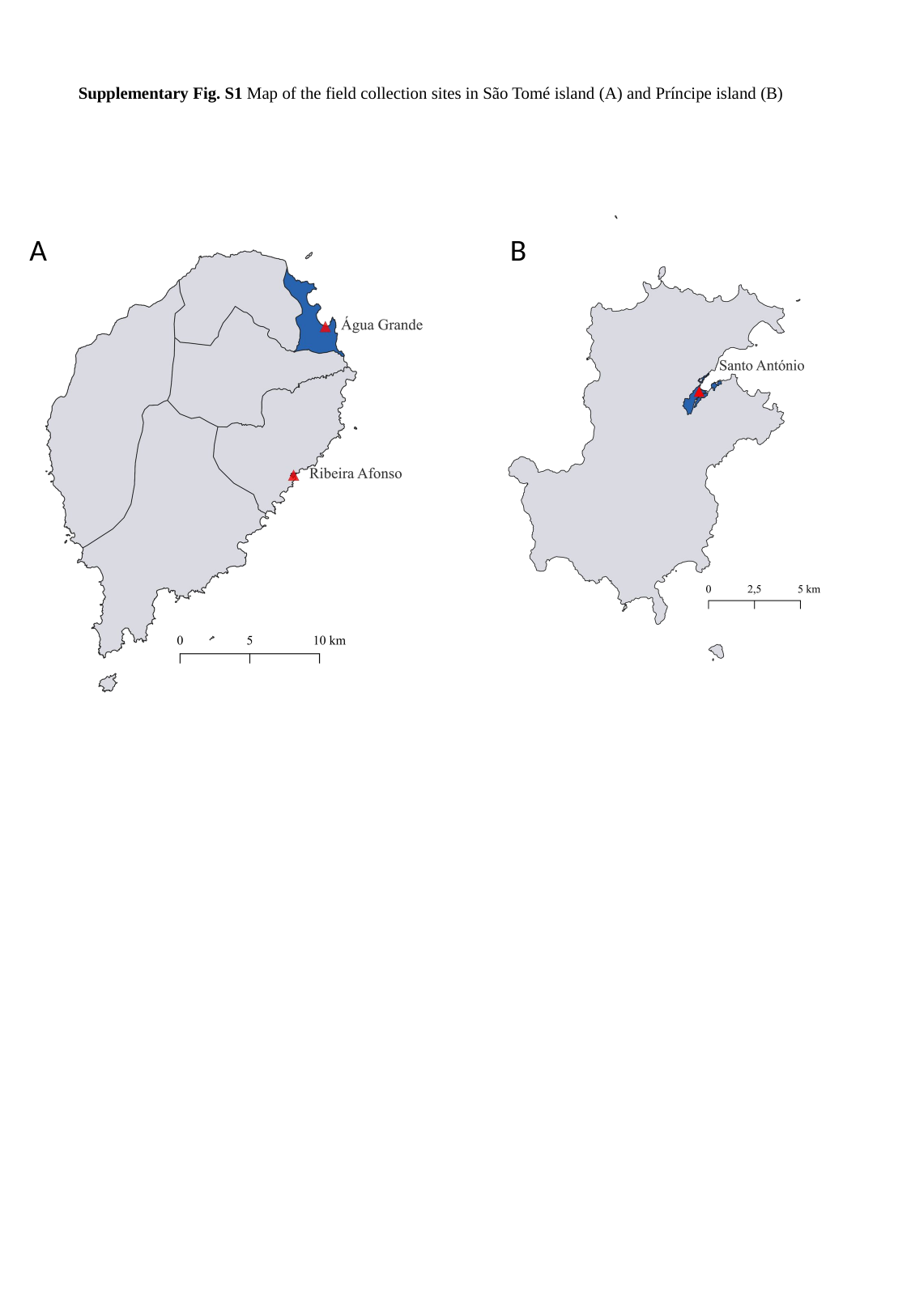

Supplementary Fig. S1 Map of the field collection sites in São Tomé island (A) and Príncipe island (B)
A
B

## Slide 2
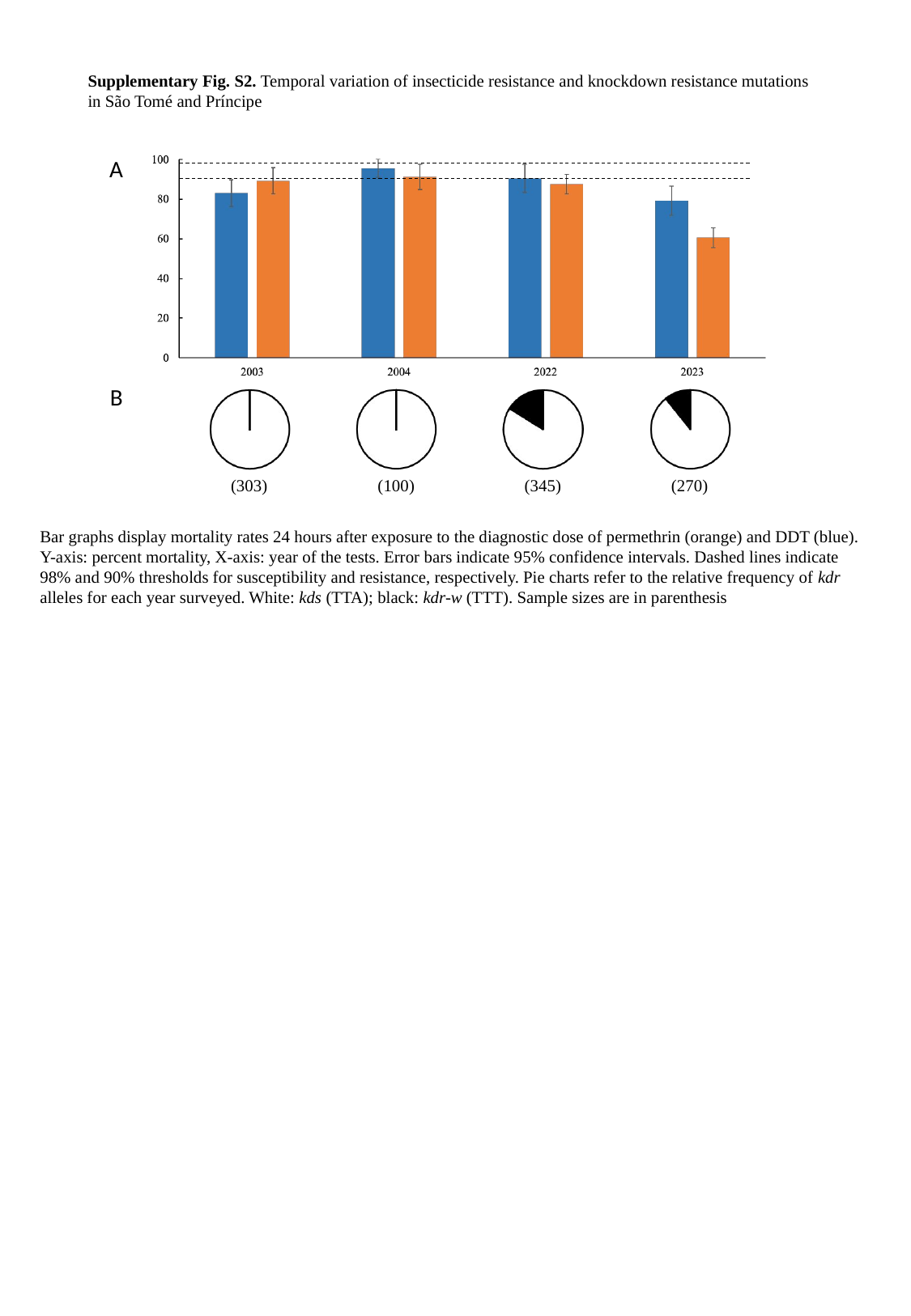

Supplementary Fig. S2. Temporal variation of insecticide resistance and knockdown resistance mutations in São Tomé and Príncipe
A
B
(303)
(100)
(345)
(270)
Bar graphs display mortality rates 24 hours after exposure to the diagnostic dose of permethrin (orange) and DDT (blue). Y-axis: percent mortality, X-axis: year of the tests. Error bars indicate 95% confidence intervals. Dashed lines indicate 98% and 90% thresholds for susceptibility and resistance, respectively. Pie charts refer to the relative frequency of kdr alleles for each year surveyed. White: kds (TTA); black: kdr-w (TTT). Sample sizes are in parenthesis
